# Multiple maxillary periodontal abscesses as a manifestation of post-coronavirus disease 2019 mucormycosis: a case report

**DOI:** 10.1186/s13256-023-03792-6

**Published:** 2023-03-03

**Authors:** Hossein Khoshkhou, Mahboube Hasheminasab, Daryoush Goudarzi Pour, Raika Jamali, Ghazal Morshedzadeh Tehrani, Neda Moslemi

**Affiliations:** 1 Department of Periodontology, School of Dentistry, Tehran University of Medical Sciences, Tehran, Iran; 2 Department of Orthodontics, Dugoni School of Dentistry, University of the Pacific, San Francisco, USA; 3 Department of Oral & Maxillofacial Radiology, School of Dentistry, Tehran University of Medical Sciences, Tehran, Iran; 4 Digestive Disease Research Institute, Tehran University of Medical Sciences, Tehran, Iran; 5 Dental Research Center, Dental Research Institute, Tehran University of Medical Sciences, Tehran, Iran; 6 Department of Periodontology, School of Dentistry, Qom University of Medical Sciences, Qom, Iran; 7 Department of Oral and Maxillofacial Surgery and CranioMaxillofacial Research Center, Tehran University of Medical Sciences, Tehran, Iran; 8 Research Development Center, Sina Hospital, Tehran University of Medical Sciences, Tehran, Iran

**Keywords:** Mucormycosis, Periodontal abscess, COVID-19, Post-COVID mucormycosis

## Abstract

**Background:**

Coronavirus disease 2019 makes patients more susceptible to superinfection of fungal disease as a consequence of immunological system impairment. Mucormycosis is a fungal infection that is rare but has a high mortality rate and mostly affects patients with poorly controlled diabetes mellitus or those receiving corticosteroids.

**Case presentation:**

Here, we present a case of post-coronavirus disease 2019 mucormycosis in a 37-year-old Persian male presenting with multiple periodontal abscess with purulent discharge and necrosis of maxillary bone (without oroantral communication). Surgical debridement following antifungal therapy was the treatment of choice.

**Conclusion:**

Early diagnosis and immediate referral are the cornerstone of comprehensive treatment.

## Background

During 2019–2022, the outbreak of coronavirus disease 2019 (COVID-19) became a universal health crisis. According to reports of the World Health Organization, by October 2021, it had caused 4,550,000 deaths worldwide. With the emergence of new variants, it seems that the pandemic will take a significant length of time to resolve. Although the majority of the patients experience mild to moderate forms of respiratory and/or gastrointestinal problems, which resolve without special medications, a number of patients develop severe forms of COVID-19 requiring medical attention.

There have been reports of the development of severe opportunistic bacterial and fungal infections in patients with COVID-19, especially in those with a history of diabetes and immune deficiency. Corticosteroids are immunosuppressants and are commonly used in patients with a serious form of COVID-19 to reduce the damage caused by the body’s immune system during severe acute respiratory syndrome coronavirus 2 (SARS-CoV-2) infection. Even in patients without diabetes, corticosteroids can increase blood sugar levels. Both factors could contribute to coinfections [1]. In addition, long-term use of antibiotics in patients with COVID-19 is considered as a predisposing factor [2].

Mucormycosis is a rare opportunistic fungal infection associated with a high rate of mortality and morbidity. The incident rate of mucormycosis varies from 0.005 to 1.7 per million in populations [3]. However, with the emergence of advanced stages of COVID-19, there is a substantial and increasing number of reports of mucormycosis in patients experiencing severe forms of COVID-19 [1]. A study in the USA showed that about 70% of patients with mucormycosis had a history of diabetes mellitus [4]. This angioinvasive infection leads to destruction of blood vessels by fungal hyphae, causing infarction and necrosis of a variety of end‐organ host tissues [1]. Rhino‐orbital cerebral infection is one of the most common clinical signs of mucormycosis. It is presumed as a secondary outcome of inhalation of spores into the paranasal sinuses in a susceptible host [5].

The most common fungal species related to mucormycosis are *Rhizopus* spp., *Mucor* spp., and *Lichtheimia* (formerly *Absidia* and *Mycocladus*) spp. Genera of other Mucorales, such as *Saksenaea*, *Rhizomucor*, *Apophysomyces*, and *Cunninghamella*, are less common. Several etiologic fungal species have been noticed from different areas. *Rhizopus* spp. is the most frequently identified species in the samples obtained from patients with mucormycosis in Europe and India [6].

Spellberg *et al.* found that infection with mucormycosis could be accompanied by fever, acute sinusitis, nasal congestion, and headache. All paranasal sinuses might be involved, and the infection may spread to nearby structure such as orbit, palate, and brain [4]. Other common signs include periorbital edema and cellulitis, ophthalmoplegia, loss of vision, and proptosis and neurological deficits [6]. Mrittika Sen *et al.* proposed a classification of rhino-orbital-cerebral mucormycosis (ROCM). In this classification, the clinical progression of mucormycosis is composed of four stages based on different compartment involvement. Nasal mucosa, paranasal sinuses, orbit, and central nervous system are the compartments involved in stages I–IV, respectively, with specified signs and symptoms [7].

At present, no definitive diagnostic method has been introduced to differentiate the symptoms of COVID-19 and fungal coinfection [8]. The major diagnostic modalities currently used for mucormycosis are direct microscopy, histopathology, and culture specimens [4].

Given that the recent pandemic is a significant public health issue globally, there needs to be a heightened awareness about mucormycosis among patients with COVID‐19. Furthermore, both conditions in combination may lead to significant morbidity and mortality. Thus, early diagnosis and prompt treatment are key to reducing morbidity. Here, we present a case of post-COVID mucormycosis represented by multiple maxillary periodontal abscesses and severe mobility of the teeth in an otherwise healthy patient.

## Case presentation

**Fig. 1:**
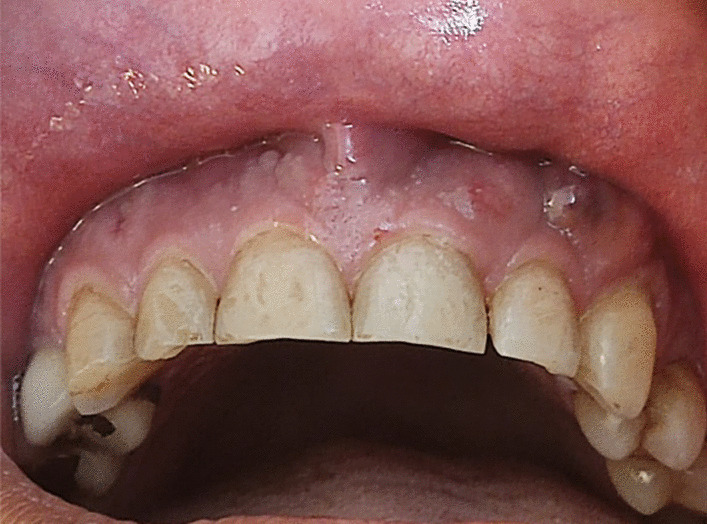
Multiple gingival abscesses in a COVID-19-positive patient with diabetes under corticosteroid therapy

A 37-year-old Persian male was referred to a private clinic of periodontics complaining about mobility of the upper teeth and purulent discharge within 1 month ago (Fig. 1). About 3 months ago, he had been hospitalized due to the severe COVID-19 with lung involvement (40%). No other systemic disease was reported, other than a long history of controlled chronic sinusitis. The patient had taken antiviral medication (remdesivir) and glucocorticosteroid (dexamethasone) in the hospital for three consecutive days. Following dexamethasone administration, serum glucose level increased up to 400 mg/dl. Thus, insulin was administered to control the high serum glucose level. However, the drug-induced diabetes could not be controlled even after administration of insulin (NovoRapid 6 units subcutaneously before lunch, Lantus 16 units subcutaneously every night, and metformin 500 mg two times daily). The patient was discharged from the hospital after resolution of COVID-19 symptoms and continued insulin injection according to the prescribed protocol.

After a few days, the patient complained of headache and pain in the maxillary region. He noticed gum swelling and gingival abscess drainage. A general dentist prescribed antibiotics [penicillin 800,000 IU intramuscularly two times a day, oral co-amoxiclav 625 mg (amoxicillin 500 mg + clavulanic acid 125 mg) three times a day, and oral metronidazole 250 mg three times a day for 1 week] and chlorhexidine 0.2% mouth rinse. No clinical improvement was noted after the administration of the antibiotics.

**Fig. 2:**
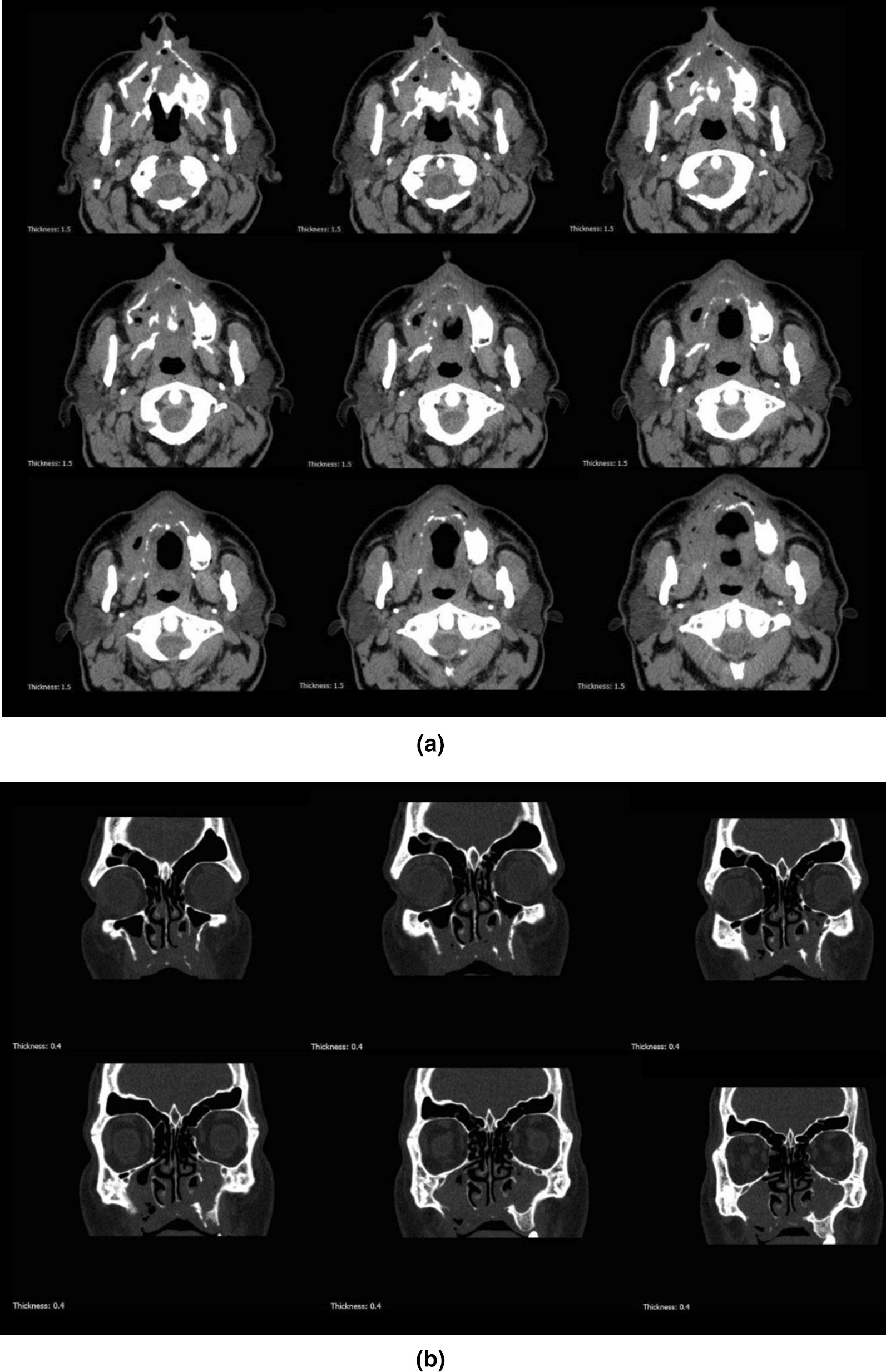
Axial (**A**) and coronal (**B**) view of computed tomography scan of the patient

The patient was referred to the ear–nose–throat (ENT) department (Amir-Alam Hospital, Tehran University of Medical Sciences) for further assessments in terms of sinus involvement. According to multidetector computed tomography (CT) images without contrast enhancement, severe and moderate opacification of right and left maxillary sinuses, respectively, and mild opacification of right ethmoidal sinus with ostiomeatal complex involvement was evident. To discover more details about the brain and para nasal sinuses, magnetic resonance imaging (MRI) with multiplanar images and pre- and post-paramagnet injection was performed. This modality showed right maxillary sinusitis with pre-antral, retro-antral, and infra-temporal soft tissue infiltration without obvious necrosis. There was bilateral nonspecific white matter high signal lesion without any orbital infiltration (Fig. 2). The ophthalmology consult revealed normal visual acuity. Therefore, at that time, there was no sign in favor of mucormycosis and “partially treated acute sinusitis” was diagnosed by the clinicians. Thus, wide-spectrum antibiotics were administered again.

**Fig. 3:**
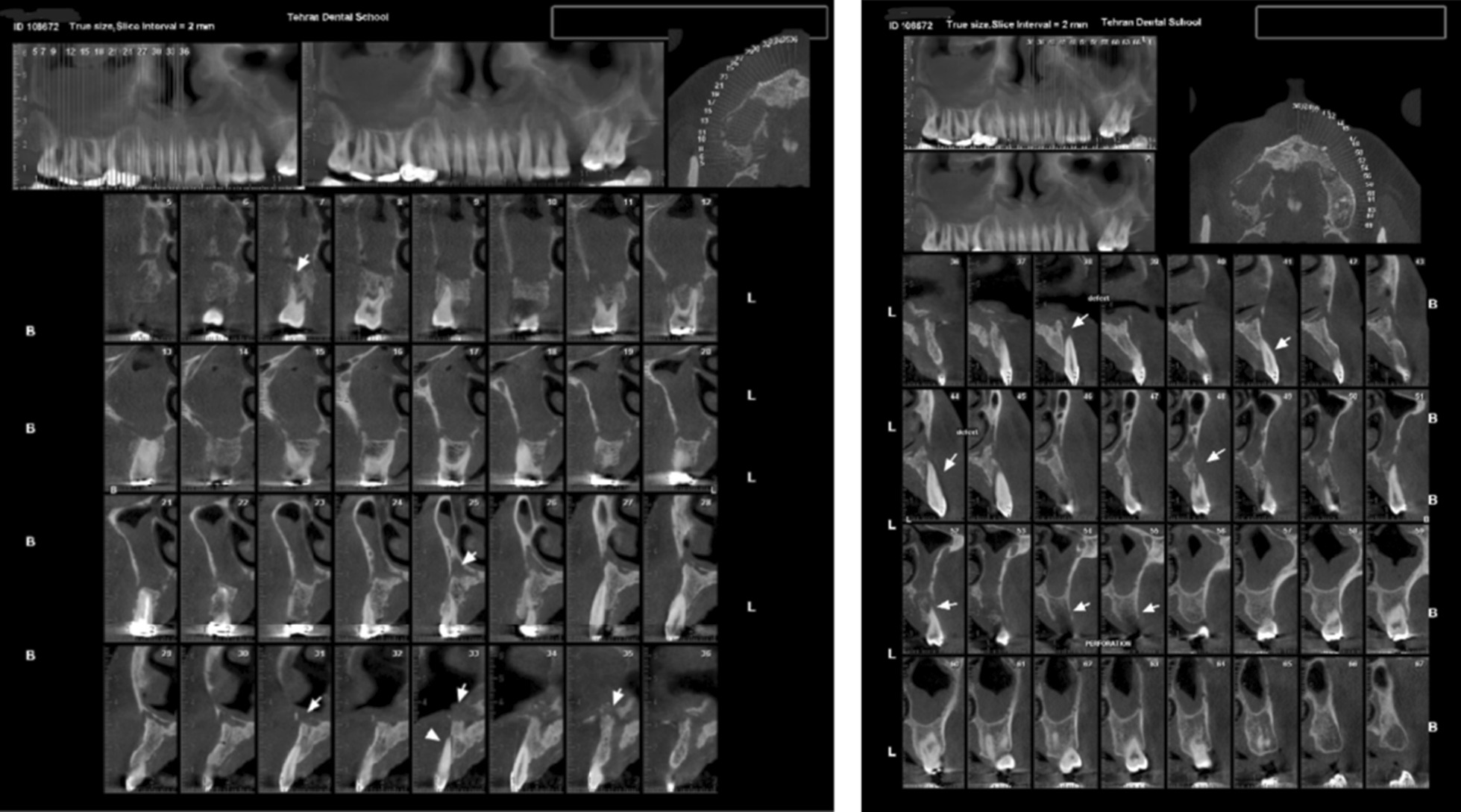
Baseline cone-beam computer tomography from right and left maxillary area

After 3 days, the patient was referred to the periodontist complaining about severe pain in the maxillary region and purulent discharge from the gingiva. Almost all maxillary teeth showed grade III mobility. Vitality test was negative for all suspected teeth. The periodontal pockets around the maxillary teeth were about 10–12 mm. There was multiple abscess and fistula tract in the apical site of all maxillary teeth. Pus discharge was noted from the gingival sulcus. Blood sugar test and cone-beam computer tomography (CBCT) from maxillary area were ordered. The blood tests showed high level blood sugar (HbA1c 10.5 and Fasting Blood Sugar 400 mg/dl). Based on the CBCT of the area, generalized rarefaction was observed from right tuberosity to left edentulous area in mesial aspect of second molar that had caused alveolar destruction and buccal and lingual defect around the teeth. Opacification was evident in both right and left maxillary sinuses. Perforation was observed in nasal floor and maxillary sinus walls, as well. Sclerosis was detected in bilateral maxillary sinus walls, due to the patient’s previous sinusitis history. Ethmoid, sphenoid, and frontal sinuses were clear (Fig. 3).

**Fig. 4:**
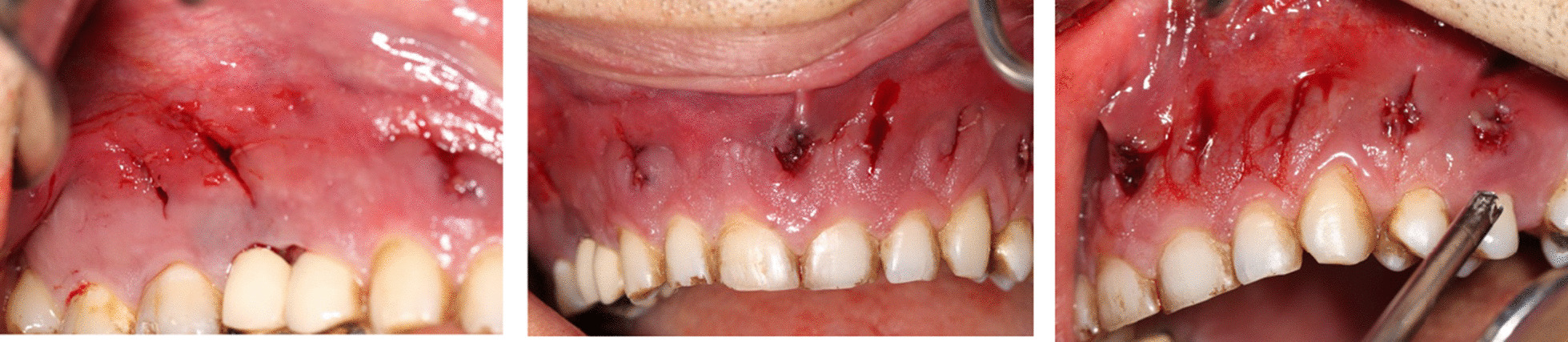
Immediately after drainage

Mucormycosis- and diabetes-related osteomyelitis were the most probable differential diagnosis. To alleviate pain, the clinician drained the abscess with curettes (Fig. 4). Despite the initial resolution and significant pain alleviation, an increasing level of pain and purulent discharge was noted during the follow-up visit. The patient was referred to a maxillofacial surgeon.

**Fig. 5:**
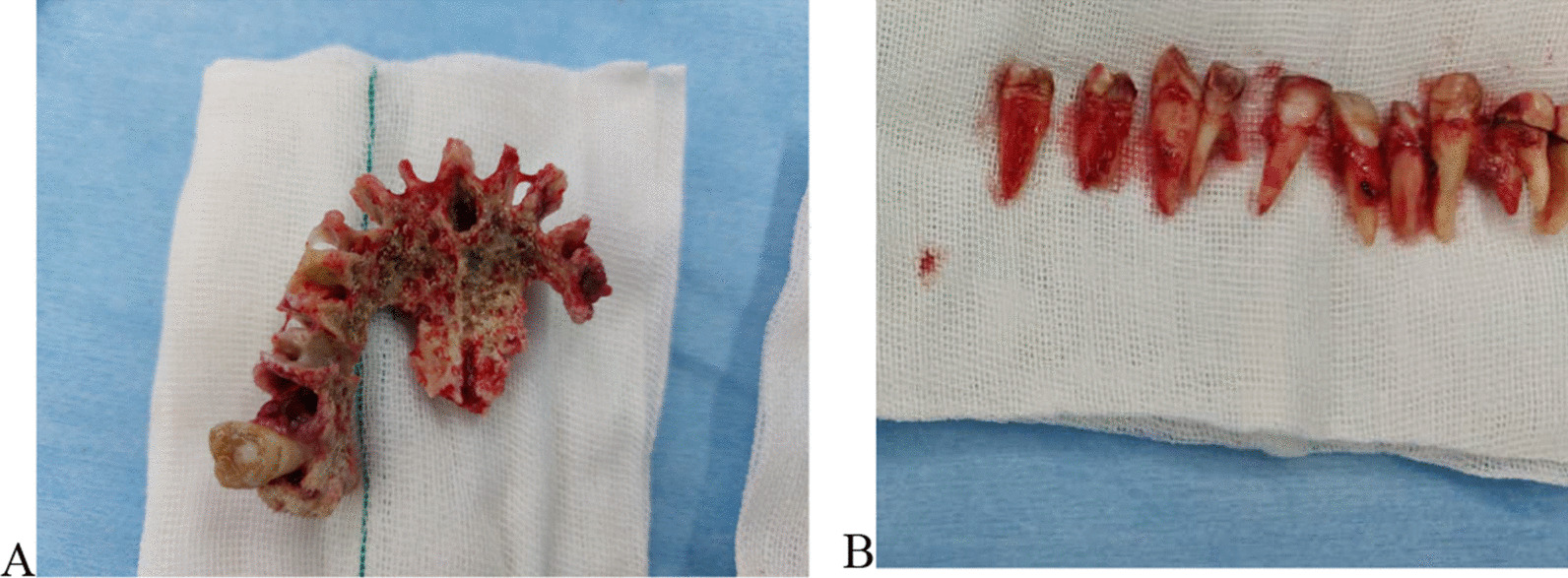
The necrotic bone (**A**) and the extracted teeth (**B**) during inferior subtotal maxillectomy

The patient was admitted at hospital. Ophthalmologist and ear–nose–throat (ENT) specialists were consulted to assess the involvement of other paranasal sinuses and orbits. Inferior subtotal maxillectomy was performed for the patient under general anesthesia, and 12 maxillary teeth were extracted (sparing both maxillary second molars). The black necrotic tissue was removed from both maxillary sinuses. The lower nasal conchae were necrotic and thus were removed on both sides (Fig. 5). The buccal and palatal soft tissue was relatively healthy, so complete closure of the defect was possible. The entire resected specimen was sent for histopathology.

According to the histopathology report, there was a portion of sinonasal tissue with severe acute-on-chronic inflammation, granulation tissue formation, presence of fibrinoleukocytic exudate, and infiltration of some multinucleated giant cells, and few small portions of dead and alive bone were also noted. Microscopic evaluation revealed the presence of rectangular-shaped aseptate hyphae in the specimens, so the diagnosis of mucormycosis was confirmed. On histopathologic analysis of right middle concha, there was no preserved fungal element in multiple cut section of hematoxylin and eosin (H&E) and periodic acid–Schiff (PAS) staining. Portions of respiratory mucosa with marked active inflammation, fibrinoleukocytic exudate, and necrosis were observed in analyzed specimen.

Immediately, antifungal therapy was started for the patient [intravenous amphotericin B (5 mg/kg/day) for 6 weeks]. Levofloxacin 500 mg (Tavanex) and nasal spray (beclomethasone) were also administered for sinusitis.

**Fig. 6:**
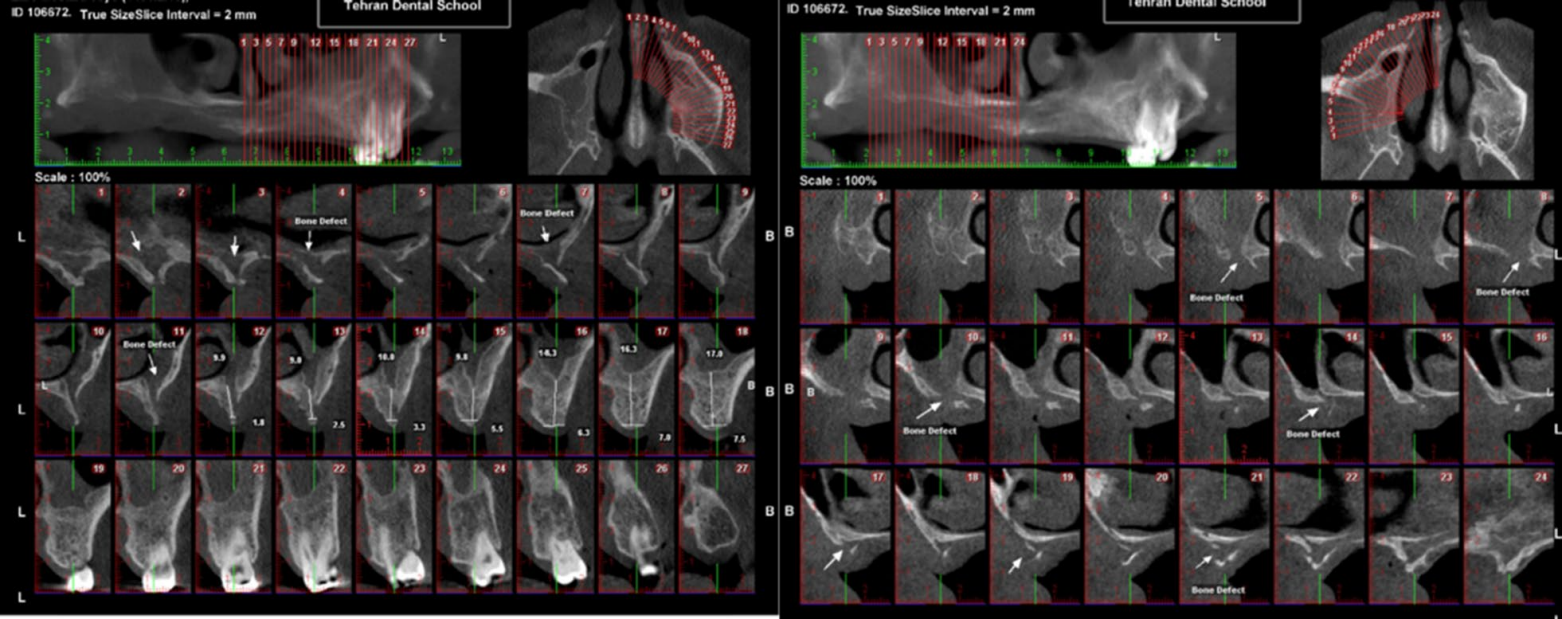
Cone-beam computed tomography after subtotal maxillectomy

**Fig. 7:**
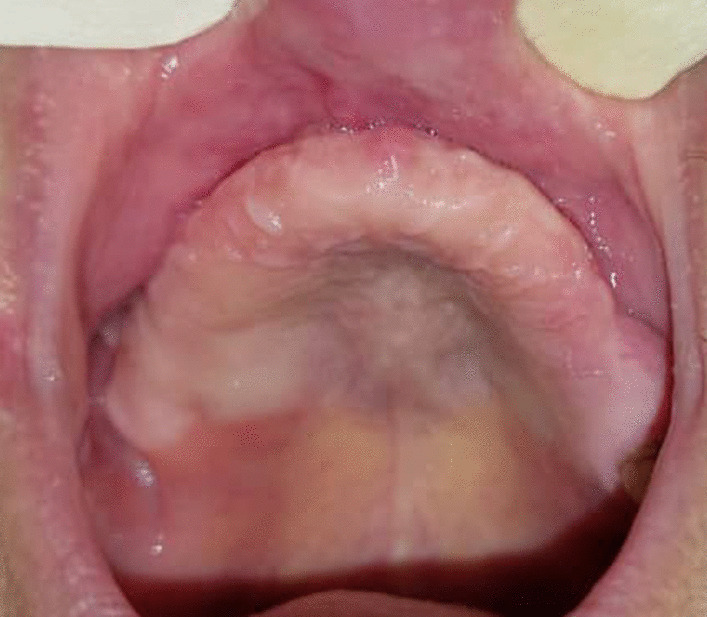
Clinical view of 2-month healing

The patient underwent multiple sinonasal endoscopy to evaluate the course of treatment. The healing was uneventful, and there was no sign of oroantral fistula. He was discharged in good condition. We followed up the patient for 6 months, and there were no signs of reinfection. The right maxillary second molar was extracted due to severe mobility (Figs. 6 and 7). Reconstruction of the residual defect with patient-specific implants is scheduled for the patient.

## Discussion

Although there are several studies reporting cases with mucormycosis following COVID-19 (Singh *et al.* 2021, Maini *et al.* 2021, Johnson *et al.* 2021, Pakdel *et al.* 2021, Buil *et al.* 2021, Arana *et al.* 2021, Ahmadikia* et al.* 2021), there are few reports about patients with post-COVID mucormycosis presenting with multiple periodontal abscess and tooth mobility. Arthanari *et al.* reported the clinical presentations of COVID-19-associated mucormycosis, multiple gingival abscess with tooth mobility, oral mucosa blackish discoloration, necrotic palatal bone, and probably extraoral swelling [9]. Moreover, in a letter to the editor, Rana *et al.* reported two post-COVID cases with transient hyperglycemia following 3 weeks of corticosteroid therapy. Thereafter, tooth mobility and periodontal abscess with palatal bone perforation were seen [10].

In a systematic review published in 2021, Singh *et al.* [11] have reported 101 patients with post-COVID-19 mucormycosis. Seventy-six percent of the patients had received corticosteroid therapy. Similarly, Pakdel and his colleagues [12], in a cross-sectional descriptive multicenter study, reported patients with COVID-19-associated rhino-orbital mucormycosis. Eighty-six percent (*n* = 13) had a history of diabetes mellitus, and 46.6% (*n* = 7) were previously under corticosteroid therapy. In their report, seven patients (47%) died because of the mucormycosis. They concluded that history of diabetes and extensive corticosteroid therapy in a patient with COVID-19 might have increased the incidence of mucormycosis. Extreme care should be exercised to keep the glucose at optimal level, and only cautious use of corticosteroid should be recommended.

In the Netherlands, a case series study reported secondary fungal infection in patients with COVID-19. Their patients showed rhino-orbital cerebral, pulmonary, and disseminated infections. Two out of four patients had poorly controlled diabetes, and both were deceased. One of the nondiabetic patients died, as well. It was concluded that extreme caution should be taken even in nondiabetic patients with no history of intensive care unit (ICU) admission [13].

In 2021, Maini *et al.* reported a patient with post COVID-19 sino-orbital mucormycosis. The patient had no history of diabetes mellitus or debilitating conditions. He presented with chemosis and pain in the left eye. The diagnosis of mucormycosis was established using functional endoscopic sinus surgery, and the patient was managed with antifungal therapy and surgical debridement. The authors suggested a prophylactic treatment protocol as well as setting up a clear guideline for less morbidity rate [14].

In the USA, Johnson *et al.* reported that combined pulmonary aspergillosis and mucormycosis could occur simultaneously as a secondary complication of COVID-19 [15].

In a case report by Ahmadikia *et al.*, steroid therapy appeared as a double-edged sword, making the patients more susceptible to invasive fungal infections. Poorly controlled diabetes mellitus and immunosuppressive conditions were considered as the predisposing factors for mucormycosis [16].

In a study of by Hosseini *et al.*, periodontal destruction, presence of oronasal/oroantral communication, and black necrotic scar and necrotic bone in the buccal vestibule, palate, and maxillary alveolus were the symptoms of ROCM. The rapid spread of mucormycosis from maxillary sinuses toward the oral cavity could be the result of angioinvasion in palatal blood vessels, which gives rises to necrotic thrombosed black-colored palate. Tooth mobility and tooth loss are the undesired consequence of bone destruction surrounding the teeth [17].

## Conclusion

Following severe forms of COVID-19, the presence of periodontal abscess with tooth mobility should be cautiously taken into consideration, especially in those with a history of corticosteroid therapy. Dentists have a crucial role in early diagnosis of rhino-maxillary mucormycosis and immediate referral to head and neck surgeons.

## Data Availability

Data will be available on request.

## Abbreviation

COVID 19: Coronavirus disease 2019
